# Case Report: Lymphohistiocytic Myocarditis With Severe Cardiogenic Shock Requiring Mechanical Cardiocirculatory Support in Multisystem Inflammatory Syndrome Following SARS-CoV-2 Infection

**DOI:** 10.3389/fcvm.2021.716198

**Published:** 2021-09-09

**Authors:** Xavier Bemtgen, Karin Klingel, Markus Hufnagel, Ales Janda, Christoph Bode, Dawid L. Staudacher, Alexander Supady, Ilona Jandova

**Affiliations:** ^1^Department of Cardiology and Angiology I (Heart Center Freiburg – Bad Krozingen), University Medical Center – University of Freiburg, Faculty of Medicine, University of Freiburg, Freiburg, Germany; ^2^Department of Medicine III (Interdisciplinary Medical Intensive Care), University Medical Center – University of Freiburg, Faculty of Medicine, University of Freiburg, Freiburg, Germany; ^3^Cardiopathology, Institute for Pathology and Neuropathology, University Hospital Tübingen, Tübingen, Germany; ^4^Department of Pediatrics and Adolescent Medicine, University Medical Center, Medical Faculty, University of Freiburg, Freiburg, Germany; ^5^Department of Pediatrics and Adolescent Medicine, Ulm University Medical Center, Ulm, Germany; ^6^Heidelberg Institute of Global Health, University of Heidelberg, Heidelberg, Germany; ^7^Department of Rheumatology and Clinical Immunology, University Medical Center, Faculty of Medicine, University of Freiburg, Freiburg, Germany

**Keywords:** COVID-19, V-A ECMO, Impella®, MIS-C, Multisystem Inflammatory Syndrome in children, myocardial biopsy

## Abstract

Multisystem Inflammatory Syndrome (MIS) is a novel hyperinflammatory syndrome associated with SARS-CoV-2 infection. It predominantly affects children (MIS-C) a few weeks after a usually asymptomatic SARS-CoV-2 infection and is only rarely seen in adults above 21 years (MIS-A). Only scarce data on histological findings in both pediatric and adult patients has been published so far. An 18-year-old male patient was admitted to hospital in a febrile state, which progressed to severe cardiogenic shock and multi-organ failure requiring extracorporeal life support. Myocardial biopsy revealed small vessel-associated immune cell infiltrates. Diagnosis of MIS-C was made after ruling out all potential differential diagnosis. Use of immunosuppressive treatment with steroids, interleukin-1 blockade and high-dose intravenous immunoglobulins resulted in the patient's full recovery. Multisystem Inflammatory Syndrome (MIS) is a new differential diagnosis of cardiac dysfunction in pediatric and adult patients. The lack of myocardial necrosis differentiates the disease from other viral myocarditis and offers an explanation for the fast response to immunomodulatory therapy and the favorable prognosis. The preceding SARS-CoV-2 infection might only have been mildly symptomatic or even asymptomatic.

## Introduction

Coronavirus disease 2019 (COVID-19) with respiratory failure is the primary complication of an infection with the severe acute respiratory syndrome coronavirus 2 (SARS-CoV-2) in adults. Here, diagnosis and treatment is progressively better understood (1). In pediatric patients however, a novel hyperinflammation syndrome called Multisystem Inflammatory Syndrome in Children (MIS-C) is a serious pathology caused by a SARS-CoV-2 infection (2). The awareness and knowledge on this hyperinflammation syndrome are steadily growing among pediatricians, but the more uncommon adult variant of this syndrome, Multisystem Inflammatory Syndrome in Adults (MIS-A), is widely unknown in adult medicine. The threshold between the pediatric and the adult variant is 21 years as defined by the CDC (3). Only scarce data on histological findings in both pediatric and adult patients has been published so far.

Here, we report the case of a young adult with severe cardiogenic shock diagnosed with severe MIS-C backed by myocardial biopsy and rapid recovery following initiation of immunosuppressive treatment.

## Case Description

**Figure 1:**
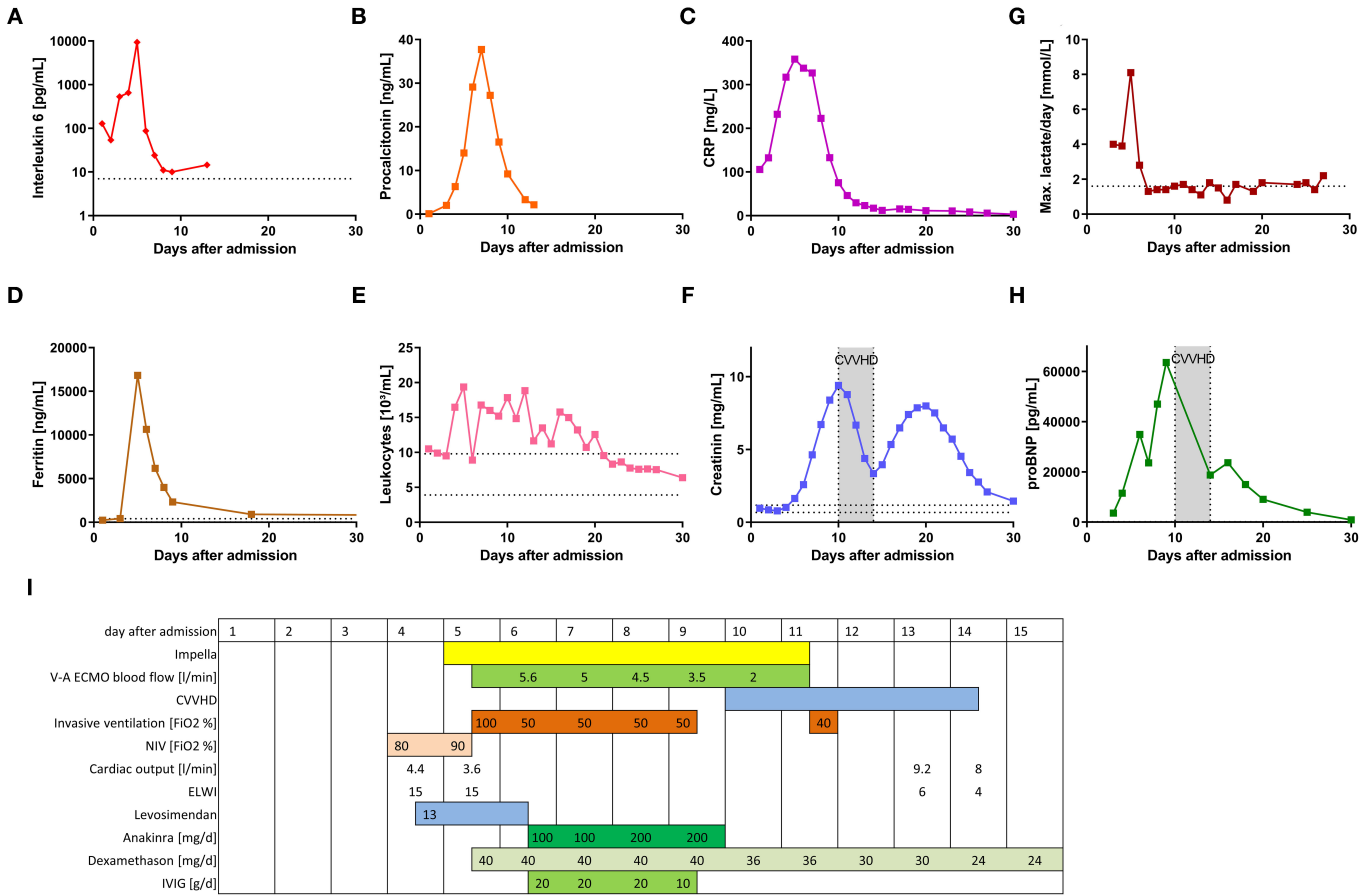
Clinical and treatment parameters of the patient during the 1st month after hospital admission. **(A–H)** show the time course of different laboratory parameters during the first 30 days following hospital admission. **(I)** displays a timeline of the different clinical parameters and specific therapy during the first 30 days.

An 18-year-old male patient presented to the emergency department with hyperpyrexia (42°C), chills and tachycardia. Physical examination and chest X-ray revealed no pathological findings. Laboratory tests showed elevated C-reactive protein (CRP; 105.9 mg/l, reference range <5 mg/l) as well as interleukin 6 serum levels (IL-6; 128 pg/ml, reference range <7 pg/ml), but only modestly elevated procalcitonin (PCT; 0.12 ng/ml, reference range < 0.05 ng/ml) (Figure 1). The patient was admitted to a standard care ward and an empiric antibiotic therapy was initiated.

The patient's medical history was unremarkable. Approximately 2 months prior to admission, the patient was exposed to Severe Acute Respiratory Syndrome Coronavirus 2 (SARS-CoV-2) and went into quarantine. A few days after this exposure, he complained he had lost his sense of smell, but he experienced no other symptoms. Neither during his quarantine nor after his initial admission to the hospital was an active SARS-CoV-2 infection ever proven, despite repeated nasopharyngeal swabs.

Following admission, the patient's condition steadily deteriorated. After 3 days he was transferred to the intensive care unit (ICU) due to arterial hypotension with suspected septic shock. Initially, intravenous fluid resuscitation and a low rate of noradrenaline (0.01 μg/kg/min) were sufficient to stabilize the patient's blood pressure. A generalized rash affecting the abdomen and all limbs occurred. On day 4 following hospital admission, transthoracic echocardiography revealed a severely impaired left ventricular cardiac function (left ventricular ejection fraction, LVEF, 25%, Supplementary Video 1). No relevant ECG pathologies were seen beside sinustachycardia. At that time, Pulse Contour Cardiac Output (PiCCO; Getinge, Rastatt, Germany) measurement confirmed marginal cardiac output of 4.4 l/min (reference range: 4–8 l/min). Computed tomography showed enlarged abdominal lymph nodes, wall thickening of the colon and polyserositis with pericardial and pleural effusions and ascites. Respiratory failure due to pulmonary edema required non-invasive ventilation (NIV).

Subsequently, on day 4 after hospital admission, liver and renal failure and massive systemic inflammatory response became evident (Figure 1). The rheumatology workup (anti-nuclear antibodies (ANA), extractable nuclear antigen (ENA), anti-neutrophil cytoplasmic antibodies (ANCA), anti-phospholipid antibodies, complement) were unremarkable. Microbiological investigation only revealed positive SARS-CoV-2 serology (anti-S1 and anti-N antibodies).

Four days after initial hospital admission, the patient was transferred to our hospital, a tertiary care center in Freiburg, Germany. Upon admission, levosimendan infusion was started. The following day, endomyocardial biopsy (EMB) was performed. Signs of hypoperfusion end organ failure persisted (elevated lactate, renal function, Figure 1). For this reason, a percutaneous ventricular assist device (Impella®, Abiomed, Danvers, NJ, United States of America) was implanted. Subsequently, cardiac output improved from 3.6 to 4.9 l/min and pulmonary capillary wedge pressure decreased from 26 to 21 mmHg.

However, due to both progressive hypoxemia under NIV with a required fraction of inspired oxygen (FiO_2_) of up to 100% and to worsening neurological symptoms (sopor), invasive mechanical ventilation was indicated. Within just a few hours after endotracheal intubation, additional venoarterial extracorporeal membrane oxygenation (V-A ECMO) support was required, because of worsening hypoperfusion and severe end-organ failure (Figure 1).

**Figure 2:**
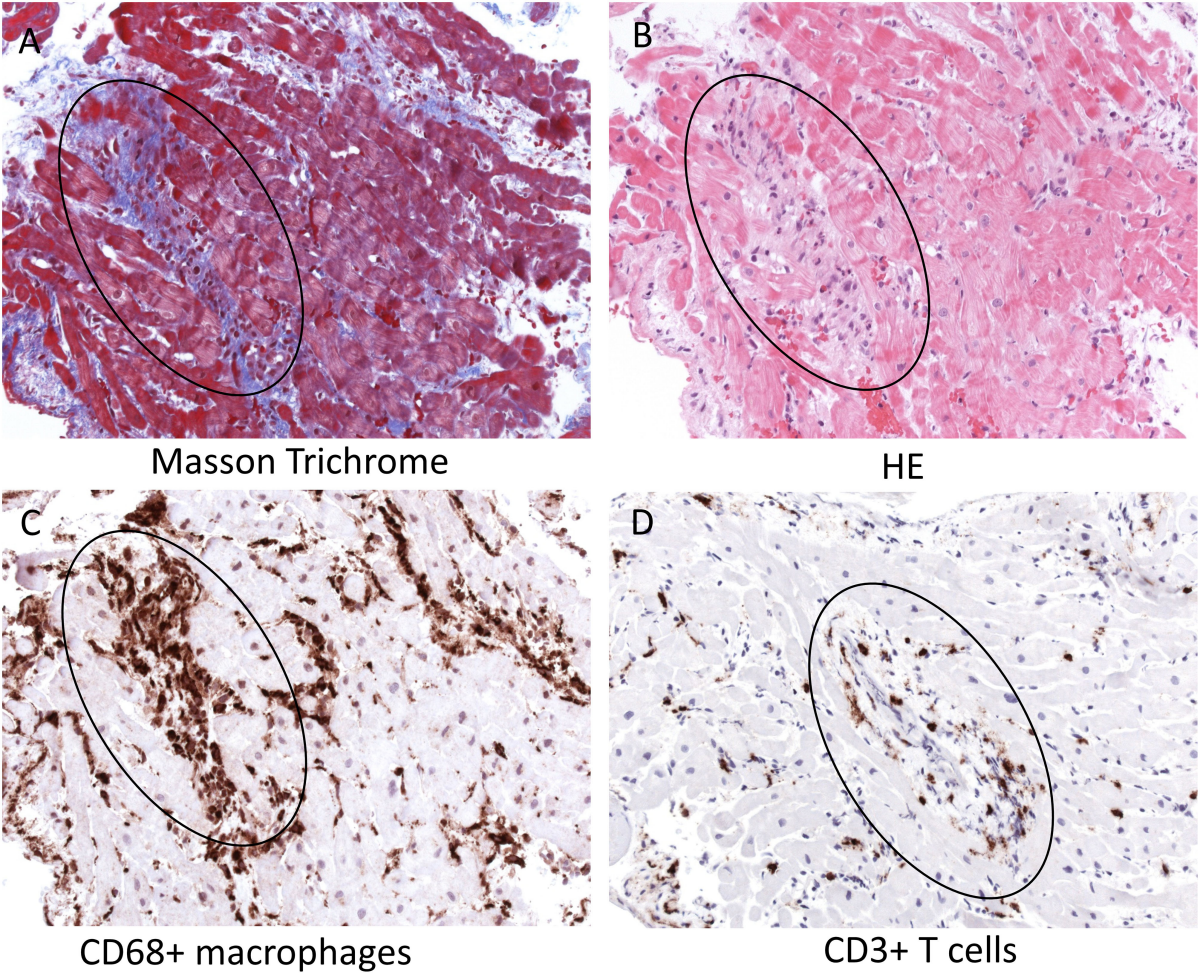
Histopathology and immunohistochemistry of the patient's endomyocardial biopsy. Serial tissue sections of paraffin-embedded endomyocardial biopsies reveal perivascular fibrosis in absence of myocyte necrosis [Masson Trichrome **(A)** and HE **(B)** stainings, see circle] and severe infiltration of CD68+ macrophages **(C)** and CD3+ T cells **(D)** primarily around intracardiac small vessels (see circle, magnification x200). HE, hematoxylin-eosin; CD, cluster of differentiation.

EMB showed a significant infiltration of immune cells into the heart. Especially CD68+ macrophages but also CD3+ T cells were found to be located primarily around small vessels within the myocardium, as shown by immunohistochemical stainings (see circle). Masson Trichrome and HE stainings further demonstrated the presence of perivascular fibrosis in serial tissue sections, but no myocyte necrosis (Figures 2A–D, see circle). Nested (RT-) PCR for the detection of enteroviruses (including coxsackieviruses of group A and B, echoviruses), parvovirus B19, human herpesvirus 6, Epstein Barr Virus, adenoviruses, human cytomegalovirus, herpes simplex virus type 1 and 2, human herpesvirus 7 (HHV7), varizella zoster virus, influenza A and B viruses, Toxoplasma gondii or borrelia spp. was negative in the myocardium and EDTA blood. In addition, qRT-PCR did not detect SARS-CoV-2 RNA in the myocardium.

Following interdisciplinary discussion (pediatrics, rheumatology, cardiology, and infectious disease), Multisystem Inflammatory Syndrome in children (MIS-C) following preceding SARS-CoV-2 infection was diagnosed and immunosuppressive therapy including high-dose intravenous immunoglobulin (IVIG), dexamethasone and IL-1-blockade (anakinra) was initiated (Figure 1). Clinical and laboratory parameters improved within 3 days and 1 day, respectively (Figure 1). As cardiac function recovered, this enabled discontinuation of extracorporeal cardiocirculatory support (V-A ECMO, Impella®) on day seven after initiation. Cardiac necrosis parameters were only moderately elevated during the shock phase (max. TroponinT 341 ng/L, ref <14 ng/L; CK-MB max 54 U/L, ref <24 U/L) indicating only a minor myocardial damage has occurred. Cardiac function did indeed fully recover (Supplementary Video 2). Renal function only was able to fully recover after 30 days to full recovery. The patient was able to be discharged 32 days after initial hospital admission.

## Discussion

Early during the SARS-CoV-2 pandemic, a novel hyperinflammatory syndrome was described. Initially, only pediatric cases were identified with symptoms and clinical findings, which in many respects resembled features of Kawasaki disease and Toxic Shock Syndrome (4, 5). Two synonymic terms—Multisystem Inflammatory Syndrome in Children (MIS-C) and Pediatric Inflammatory Multisystem Syndrome temporarily associated with SARS-CoV-2 (PIMS-TS)—were established (3, 6, 7). Later, a similar syndrome was reported in adults (MIS-A) (8, 9).

Our patient fulfilled the diagnostic criteria of MIS-C with fever, rash, lymphadenopathy, shock, myocardial injury, colitis, and positive SARS-CoV-2 serology, as well as severe inflammatory response. For this reason, immunomodulatory therapy based on clinical recommendations of the American College of Rheumatology (ACR) was initiated (3).

ACR recommends steroid treatment with methylprednisolone (20–30 mg/kg a day, for 1–3 days up to 1 g per day followed by tapering doses−2 mg/kg a day, maximum 60 mg a day); high-dose intravenous immune globulin therapy (2 g/kg a dose) in moderate to severe cases; and cytokine receptor (IL-1 or IL-6) blockade (3).

As with myocardial involvement following other viral infections, cardiac injury in MIS-C may occur either due to direct cardiac invasion by the virus (10–13) or else following accompanying cytokine storm (2). Since EMB is only rarely performed, the reported cases of myocardial injury in the context of the SARS-CoV-2 infection are largely based upon clinical symptoms, laboratory results and imaging findings (e.g., electro- and echocardiography, magnetic resonance). Arrhythmias, decreased LVEF and high prevalence of cardiogenic shock were reported (14). Histopathological investigations of EMB in patients with COVID-19 revealed multi-focal lymphocytic and interstitial macrophage infiltrates (15) without substantial myocyte necrosis. Despite the fact that SARS-CoV-2 can infect macrophages but also myocytes (16), this virus is obviously not cytolytic as e.g., coxsackievirus B3 (17). So far, the exact molecular mechanisms by which the infiltration of many macrophages and less T cells are induced in MIS patients are not known. It is likely that SARS-CoV-2 rather induces an inflammatory response by cytokine release, thus resulting in a kind of indirect myocardial injury (18). Further investigations are required to investigate why in MIS patients but not in other patients with myocardial SARS-CoV-2 infections the inflammation is associated with the vessels (19). It has to be discussed whether the presence of extensive perivascular lympho-histiocytic infiltrates without myocyte necrosis may explain the rapid response to immunosuppressive therapy in our patient. This is similar to other reported cases (9, 14, 20).

## Conclusion

Even following asymptomatic SARS-CoV-2 infection, children and young adults may develop severe Multisystem Inflammatory Syndrome in Children or Adults (MIS-C/A). In our case report, myocardial involvement (verified by endomyocardial biopsy) caused severe cardiogenic shock requiring medical as well as mechanical cardiocirculatory support. Early immunomodulatory treatment with glucocorticoids, intravenous immunoglobulin and cytokine receptor blockade helped control symptoms and interrupt uncontrolled inflammatory response. The patient's cardiac function recovered after 7 days on mechanical cardiocirculatory support with Impella® and V-A ECMO. Prompt diagnosis of MIS-C is critical, as swift use of intense immunosuppressive therapy may lead to a better prognosis for the patient. Therefore, we advise critical care clinicians to consider this differential diagnosis early on when confronted with patients suffering from severe inflammatory response and impaired cardiac function.

## Data Availability Statement

The original contributions presented in the study are included in the article/Supplementary Material, further inquiries can be directed to the corresponding author/s.

## Ethics Statement

Written informed consent was obtained from the individual(s) for the publication of any potentially identifiable images or data included in this article.

## Author Contributions

XB, AS, and IJ conceived and designed the case report, collected the data, and wrote the manuscript. KK contributed to the pathology diagnosis. IJ, MH, DS, and AJ contributed to the clinical diagnosis. CB supervised the conception, analysis, design of the work, and manuscript drafting. All authors critically revised the manuscript for important intellectual content and provided approval of the final version.

## Funding

This work was supported by Deutsche Herzstiftung COVID-19. The article processing charge was funded by the Baden- Wuerttemberg Ministry of Science, Research and Art and the University of Freiburg in the funding programme Open Access Publishing.

## Conflict of Interest

AS reports research grants and lecture fees from CytoSorbents and lecture fees from Abiomed, both outside the submitted work. The remaining authors declare that the research was conducted in the absence of any commercial or financial relationships that could be construed as a potential conflict of interest.

## Publisher's Note

All claims expressed in this article are solely those of the authors and do not necessarily represent those of their affiliated organizations, or those of the publisher, the editors and the reviewers. Any product that may be evaluated in this article, or claim that may be made by its manufacturer, is not guaranteed or endorsed by the publisher.

## Supplementary Material

Supplementary Video 1 Apical 4 chamber view at admission. Severely impaired left ventricular function seen directly after admission to our hospital.

Supplementary Video 2 Apical 4 Chamber view after recovery. Fully recovered function seen 2 days after V-A ECMO and Impella® explantation.

## Abbreviations

ACR: American College of Rheumatology
ANA: Anti-nuclear antibody
ANCA: Anti-neutrophil cytoplasmic antibodies
CD: cluster of differentiation
CDC: Centers for Disease Control and Prevention
CRP: C-reactive protein
EMB: endomyocardial biopsy
ENA: Extractable nuclear antigen
FiO_2_: fraction of inspired oxygen
HE: hematoxylin-eosin
ICU: Intensive care unit
IL-1: interleukin 1
IL-6: interleukin 6
IVIG: intravenous immunoglobulin
LVEF: left ventricular ejection fraction
MIS-C: Multisystem Inflammatory Syndrome in Children
MIS-A: Multisystem Inflammatory Syndrome in Adults
NIV: non-invasive ventilation
PCT: procalcitonin
PiCCO: Pulse Contour Cardiac Output
PIMS-TS: Pediatric Inflammatory Multisystem Syndrome temporarily associated with SARS-CoV-2
SARS-CoV-2: severe acute respiratory syndrome coronavirus 2
V-A ECMO: venoarterial extracorporeal membrane oxygenation
WHO: World Health Organization.
